# Effect of Arsenate and p-Phenylenediamine on the Expression of Aquaporins in Cultured Human Urothelial Cells

**DOI:** 10.7759/cureus.43606

**Published:** 2023-08-16

**Authors:** Yi-Hsiao Wu, Kuan-Hung Lai, Chienn-Chung Chen, Tung-Mao Lai, Po-Wei Huang

**Affiliations:** 1 Division of Cardiovascular Surgery, Department of Surgery, Zuoying Branch of Kaohsiung Armed Forces General Hospital, Kaohsiung, TWN; 2 Division of Plastic Surgery, Department of Surgery, Zuoying Branch of Kaohsiung Armed Forces General Hospital, Kaohsiung, TWN; 3 Department of Plastic Surgery, E-DA Hospital, Kaohsiung, TWN; 4 Department of Plastic Surgery, E-Da Hospital, Kaohsiung, TWN; 5 Center of General Education, Shu Zen Junior College of Medicine and Management, Kaohsiung, TWN; 6 Division of Urology, Department of Surgery, Zuoying Branch of Kaohsiung Armed Forces General Hospital, Kaohsiung, TWN

**Keywords:** urinary bladder, urothelial cell, aquaporin, p-phenylenediamine, arsenate

## Abstract

Background: Exposure to arsenic (As) or *p*‑phenylenediamine (PPD) can lead to dysfunction, or even cancer, in various types of organs, including the urinary bladder, yet the underlying mechanisms remain unclear. Aquaporins (AQPs) are widely expressed small water channel proteins that provide the major route for the transport of water and other small molecules across plasma membranes in diverse cell types. Altered expression of AQPs has been associated with pathologies in all major organs, including the urinary bladder.

Objective: The present in vitro study was performed as a first step towards exploring the possible involvement of AQPs in As- and PPD‑induced bladder diseases.

Methods: An immortalized normal human urothelial cell line was employed. Cells were exposed to different concentrations of sodium arsenate (0‑20 μM) or PPD (0‑200 μM) for 48 h. Cell viability was subsequently assessed. The mRNA and protein expression levels of AQPs (specifically, AQP3, 4, 7, 9, and 11) were analyzed using reverse transcription‑quantitative polymerase chain reaction and Western blot analyses, respectively.

Results: The viability of the cells was decreased in a concentration-dependent manner upon exposure to arsenate. The mRNA and protein expression levels of AQP3, 4, 7, and 9 were substantially reduced, whereas the expression of AQP11 was largely unchanged. As for the experiments with PPD, treatment with increasing concentrations of PPD induced a gradual decrease in cell viability. The mRNA and protein expression levels of AQP3, 4, and 11 were generally unaltered; however, a marked reduction in the expression levels of AQP7 was observed, contrasting with a gradual concentration-dependent decrease in the expression of AQP9.

Conclusion: The importance of the differential expression profiles of the AQPs induced by arsenate and PPD requires further investigation; nevertheless, the findings of the present study suggest that AQPs have a role in As‑ and PPD‑induced bladder diseases.

## Introduction

Arsenic (As) is the most prevalent environmental toxicant, and humans are exposed to As predominantly from food and water supplies. Trivalent arsenite (As^III^) and pentavalent arsenate (As^V^) compounds are the major inorganic forms that are toxic for humans, causing various diseases, including urinary bladder dysfunction, or even cancer [1,2]. As^V^ is taken up into most cells via phosphate-uptake systems, whereas entry of As^III^ into cells is primarily mediated by aquaglyceroporins, a subset of the aquaporin (AQP) family of proteins (namely, AQPs 3, 7, 9, and 10), and glucose permeases [3,4]. Inside the cells, As^V^ is reduced to the more toxic As^III^, the majority of which is metabolized first to monomethylarsonic acid (MMA^V^) and subsequently to dimethylarsinic acid (DMA^V^). A proportion of the inorganic arsenicals, together with their methylated metabolites, are excreted in urine mainly through AQPs and glucose transporters. Prior to their elimination, an accumulation of As and its toxic metabolites occurs in the bladder, where they are generally hypothesized to produce reactive oxygen species (ROS), which results in cytotoxicity, oxidative stress, altered DNA repair, microRNA dysregulation, and so on [5].

Para-phenylenediamine (PPD) is an aromatic amine that is widely used in industrial manufacturing and commercial oxidative-type hair dyes [6,7]. PPD is metabolized into the cytotoxic compound, quinonediamine. Detoxification is achieved via acetylation into the major metabolites, N‑acetyl‑p‑phenylenediamine (MAPPD) and N,N’-diacetyl-p-phenylenediamine (DAPPD), for urinary excretion [8,9]. Previous studies have demonstrated that PPD and its toxic metabolites could lead to bladder dysfunction by inducing an excessive production of ROS, and through other undefined mechanisms [10,11].

AQPs are a family of homologous water channel proteins with molecular weights ranging between 25 and 34 kDa that provide a major route for osmotically driven water movement across the plasma membrane in various cell types. To date, at least 13 distinct subtypes of AQPs (AQPs 0‑12) have been identified, which are functionally subdivided into orthodox AQPs (AQP0, 1, 2, 4, 5, 6, and 8, which are primarily water selective), aquaglyceroporins (AQP3, 7, 9 and 10, which are permeable to water and neutral solutes such as glycerol and urea) and superaquaporins (AQP11 and 12, for which details of their localization and functions remain undetermined) [12,13]. Another subclass of the AQP family, the peroxiporins (AQP1, 3, 5, 8, 9, and 11, which are permeable to hydrogen peroxide), has been recognized in recent years [14,15]. AQP research has lately been gaining a lot of additional attention, courtesy of the physiological importance of AQPs and the fact that they have been implicated in a wide range of disorders, including those affecting the urinary bladder [16].

In the human urothelium, which presents a permeability barrier lining the bladder and the associated urinary tract, the presence of five AQP subtypes (AQP3, 4, 7, 9, and 11) has been confirmed both at the mRNA and at the protein levels [17]. Although the precise functional significance of their presence has yet to be fully elucidated, the important role exerted by AQPs on the regulation of the urothelial cell volume and determination of the final urine composition cannot be underestimated. Such a role for the AQPs has been demonstrated in the adult mammalian kidney and in the skin and urinary bladder of amphibians [18-20]. Recent studies have demonstrated alterations in the expression levels of AQPs in the urothelium and lamina propria of the adult mammalian bladder following urothelial carcinoma, bladder outlet obstruction, or dehydration, emphasizing the need for an improved understanding of the role of AQPs in the urinary tract [16,21]. The present in vitro study was therefore performed as a first step towards exploring the possible involvement of AQPs in As‑ and PPD‑induced bladder diseases, and findings from this study may indicate the role of AQPs in this aspect.

## Materials and methods

Cell culture and treatment

Human urinary tract epithelial cells (SV‑HUC‑1; BCRC no. 60358), an SV40‑immortalized normal human urothelial cell line, were purchased from Bioresource Collection & Research Center (Taiwan) and cultivated in Ham’s F12 medium (Sigma‑Aldrich) containing 7% fetal bovine serum (Life Technologies; Thermo Fisher Scientific, Inc.). Cells were seeded in 10 cm dishes (for mRNA and protein expression experiments) or 96‑well plates (for cell viability assay) and allowed to grow to confluence. Cells were treated with different concentrations (0, 5, 10, 20 μM) of sodium arsenate (dibasic heptahydrate, Sigma‑Aldrich) and then incubated for 48 h. PPD (Sigma‑Aldrich) was dissolved in DMSO to prepare 0.2 M stock solution, and the maximum final concentration of DMSO in the culture medium did not exceed 0. 1% (v/v). The final concentrations of PPD were 50, 100 and 200 µM.

3‑(4, 5‑dimethylthianol‑2‑yl)‑2, 5 diphenyltetrazoliumbromide (MTT) reduction assay

This cell viability assay is based upon the capacity of mitochondrial enzymes to transform MTT to MTT formazan. Briefly, cells were seeded in 96‑well plates, allowed to grow to confluence, and then treated with various concentrations (0, 5, 10, and 20 µM) of sodium arsenate or (0, 50, 100, and 200 µM) of PPD for 48 h. The culture medium was then removed and MTT solution (5 mg/mL in PBS) was added to each well to a final concentration of 0.5 mg/mL. Following incubation at 37°C for a period of 2 h, the medium was aspirated and an equal volume of dimethyl sulfoxide (DMSO) was added to each well in order to dissolve the reduced MTT formazan crystals. The absorbance of the color product at 570 nm against the 630 nm reference was measured using a microplate reader.

Real‑time quantitative polymerase chain reaction (RT‑qPCR)

Total RNA was extracted from cells using a TRIzol Plus RNA Purification Kit (Invitrogen; Thermo Fisher Scientific, Inc.). First‑strand cDNA synthesis was performed using 2 ng of total RNA and a QuantiTect Reverse Transcription Kit (Qiagen). Real‑time PCR amplification was performed, according to the manufacturer’s protocol, on a thermal cycler (ABI 7500, Applied Biosystems, Thermo Fisher Scientific, Inc.) using a Maxima SYBR Green/ROX qPCR Master Mix (2x) (Thermo Scientific). Thermal cycling was performed as follows: denaturation at 94°C for 30 s, annealing at 56°C for 40 s, and extension at 72°C for 60 s. The primers used were as follows: AQP3 forward, 5'‑CCCGCCATGGGTCGACAGA- AGGAG‑3' and reverse, 5'‑CACTCAGATCTGCTCCTTGTGCTT‑3'; AQP4 forward, 5'‑GCACCAGGAAGATCAG CATCG‑3' and reverse, 5'‑CAGGTCATCCGTCTCTACCT-GCCTG‑3'; AQP7 forward, 5'‑ATCTCTGGAGCCCACATGAA‑3' and reverse, 5'‑GAA- GGAGCCCAGGAACTG‑3'; AQP9 forward, 5'‑TCCGAACCAAGCTTCGTATC‑3' and reverse, 5'‑GGTTGATGTGAC CACCAGAG‑3'; AQP11 forward, 5'‑TCTCTGAGTT- CTTGGGCACG‑3' and reverse, 5'‑TAGCGAAAGTGCCAAAGCTG‑3'; GAPDH forward, 5'‑AGCCACATCGCTC AGACA‑3' and reverse, 5'‑GCCCAATACGACCAAATCC‑3'). The expression levels of all mRNAs normalized to GAPDH were quantitatively analyzed according to the relative quantitation comparative Ct (2^-ΔΔ^Cq) method [22].

Western blotting

Cells were harvested in ice‑cold RIPA lysis buffer (cat. no. 89900; ThermoFisher Scientific, Inc.) containing protease inhibitor cocktail (Sigma‑Aldrich; Merck KGaA), incubated on ice for 15 min, centrifuged (15000 xg, 10 min) and then the protein concentrations of supernatants were determined using a Bradford protein assay kit (BIO‑RAD). Proteins (60 μg) were separated by 12% sodium dodecylsulfate polyacrylamide gel electrophoresis and then transferred to hybond‐polyvinylidene difluoride membranes. After 3 h of blocking with Tris‑buffered saline containing 5% nonfat dry milk and 0.1% Tween 20, the membranes were incubated overnight with polyclonal antibodies (from Alpha Diagnostic Inc.) to AQP3 (1 µg/ml; cat. no. AQP31‑A), AQP 4 (1 µg/ml; cat. no. AQP41‑A), AQP7 (1 µg/ml; cat. no. AQP71‑A), AQP9 (1 µg/ml; cat. no. AQP91‑A), and AQP11 (1 µg/ml; cat. no. AQP115‑A), or β‑actin (1:1,000; MAB1501, EMD Millipore), diluted in the blocking solution. The membranes were washed and incubated with horseradish peroxidase‑conjugated secondary antibodies (1:10,000; cat. no. ab97046, Abcam) for 1 h. The protein expression levels were detected by an enhanced chemiluminescence detection kit (Amersham) and the signal was captured by a UVP BioSpectrum500 imaging system (UVP).

Data analysis

Results were presented as the mean ± standard error, representative of 3‑5 experimental repeats. Differences between groups were assessed using one‑way ANOVA followed by Bonferroni’s test using SigmaPlot 13 (Systat Software). A p < 0.05 was considered significant.

## Results

Impact of arsenate on SV‑HUC‑1 cell viability

**Figure 1:**
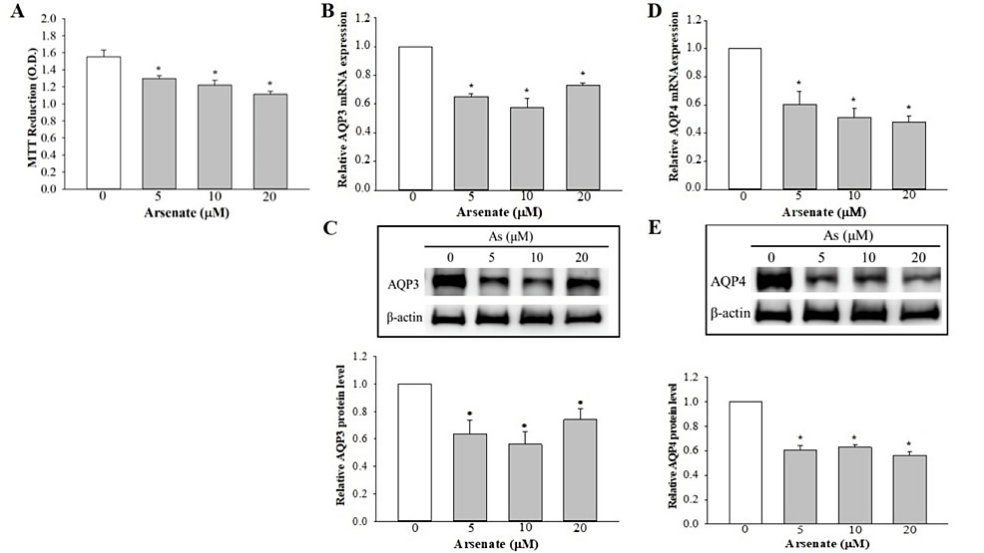
Effect of sodium arsenate on the cell viability and expression levels of AQP3 and AQP4 in SV-HUC1 cells. (A) Cell viability. The data are presented as the mean ± SEM for three independent experiments. (B, D) RT‑qPCR analysis was used to examine the effect of arsenate on the mRNA expression levels of AQP3 and AQP4. The results are shown as the mean ± SEM for data from four different experiments, and the expression levels were normalized against that of the housekeeping gene, GAPDH. (C, E) Western blot analysis was used to detect the effect of arsenate on the protein expression levels of AQP3 and AQP4. The expression levels were normalized against that of β‑actin. *P<0.05 compared with control (0 µM As). AQP: aquaporin, SEM: standard error of the mean, RT-qPCR: reverse transcription-quantitative polymerase chain reaction

The viability of urothelial cells treated with arsenate for 48 h was evaluated by MTT assay. As shown in Figure 1A, treatment with 5 μM arsenate induced an approx. 20% reduction in cell viability, followed by a further gradual decrease in cell viability in cells exposed to higher concentrations of arsenate.

Effect of arsenate on the expression of AQP3 and AQP4

At the transcript level, the expression of AQP3 was inhibited by approx. 35% following the addition of 5 μM arsenate (Figure 1B). A further small decrease in expression was seen in cells exposed to 10 μM arsenate, whereas a slight increase was detected in cells treated with 20 μM arsenate. However, no significant differences in the expression levels of AQP3 were observed among the As‑exposed cell groups. The results of the Western blot analysis confirmed the findings with the RT‑qPCR experiments, showing a moderate reduction in AQP3 expression in the As‑treated cells (Figure 1C). Similar observations were also made regarding the expression level of AQP4 mRNA and protein (Figures 1D, 1E).

Arsenate inhibits the expression of AQP7 and AQP9

**Figure 2:**
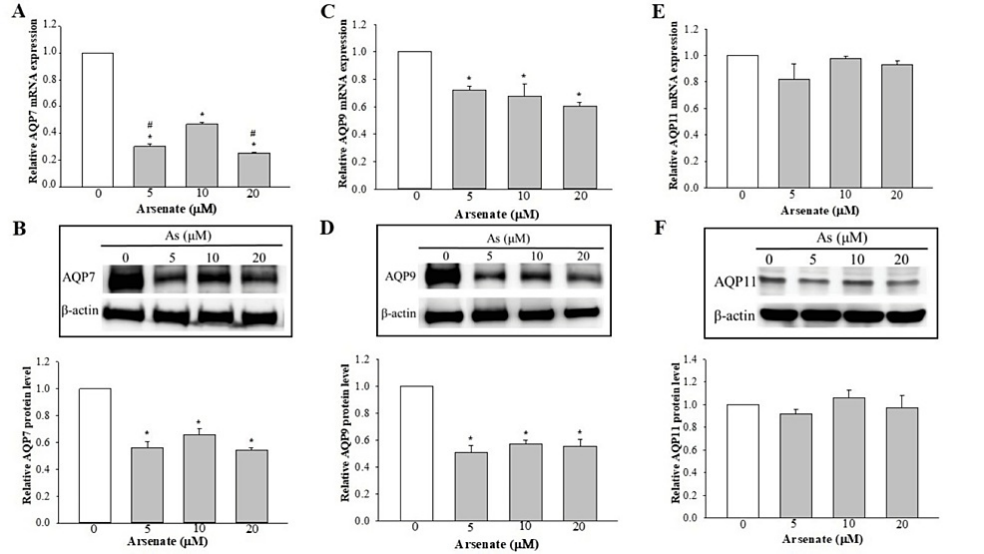
Effect of sodium arsenate on the expression levels of AQP7, 9, and 11 in SV-HUC1 cells. (A, C, E) RT‑qPCR analysis of AQP7, AQP9, and AQP11. The results are shown as the mean ± SEM for data from four different experiments, and the expression levels were normalized against that of the housekeeping gene, GAPDH. (B, D, F) Western blot analysis was used to detect the protein expression levels of AQP7, AQP9, and AQP11. The expression level was normalized against that of β‑actin. *P<0.05 vs. control (0 µM As); #P<0.05 vs. 10 µM. AQP: aquaporin, SEM: standard error of the mean, RT-qPCR: reverse transcription-quantitative polymerase chain reaction

Compared with the expression level of the control, a much lower expression level of AQP7 mRNA was observed in As-treated groups, and cells treated with 10 μM arsenate showed a significantly higher expression level than other groups (Figure 2A). These observations were corroborated by the results of the protein expression levels revealed in the Western blot analysis, albeit no significant differences in the expression levels were observed among the As‑exposed groups (Figure 2B). With respect to AQP9, arsenate caused a significant decrease in the expression level of AQP9 at both the transcript and the protein levels (Figures 2C, 2D).

The expression of AQP11 is unaltered by arsenate

The results of the RT‑qPCR analysis revealed that treatment with arsenate at various concentrations did not significantly affect the expression of the AQP11 transcript (Figure 2E). Similarly, regarding the protein expression levels, no detectable differences in the expression levels among the control and the As-exposed groups were observed (Figure 2F).

Impact of PPD on SV‑HUC‑1 cell viability

**Figure 3:**
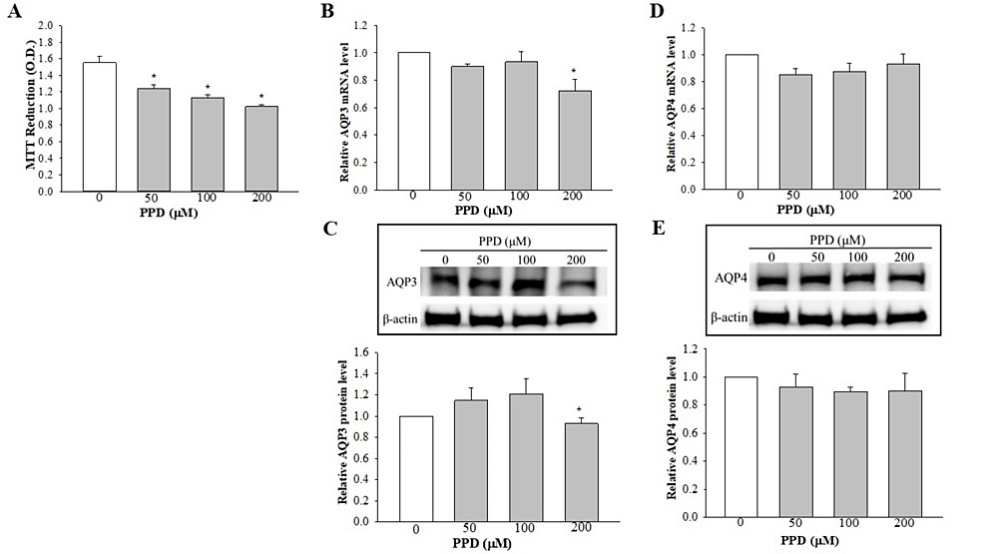
Effect of PPD on the cell viability and expression levels of AQP3 and AQP4 in SV-HUC1 cells. (A) Cell viability. Data are shown as the mean ± SEM of three independent experiments. (B, D) RT‑qPCR analysis was used to examine the effect of PPD on the mRNA expression levels of AQP3 and AQP4. The results shown are the mean ± SEM for data from four different experiments, and the expression levels were normalized against that of the housekeeping gene, GAPDH. (C, E) Western blot analysis was used to detect the effect of PPD on the protein expression levels of AQP3 and AQP4. The expression levels were normalized against that of β‑actin. *P<0.05 vs. control (0 µM PPD). AQP: aquaporin, SEM: standard error of the mean, RT-qPCR: reverse transcription-quantitative polymerase chain reaction, PPD: p-phenylenediamine

The viability of the SV‑HUC1 cells was observed to be reduced by PPD in a concentration‑dependent manner (Figure 3A).

Effect of PPD on the expression levels of AQP3 and AQP4

As shown in Figure 3B, treatment with PPD at concentrations of 50 or 100 μM did not alter the expression level of the AQP3 transcript, whereas a significant decrease in expression was observed using 200 μM PPD. Similar expression profiles were obtained upon investigating the protein expression levels of PPD (Figure 3C). With respect to AQP4, treatment with PPD did not elicit any significant changes in the expression levels of AQP4 at either the transcript or the protein level (Figures 3D, 3E).

Effect of PPD on the expression levels of AQP7 and AQP9

**Figure 4:**
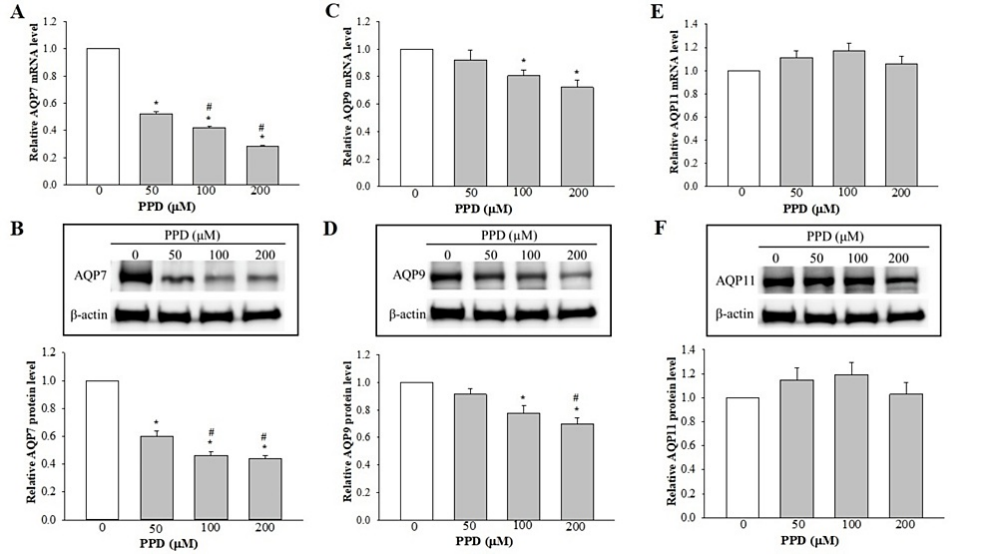
Effect of PPD on the expression levels of AQP7, 9, and 11 in SV-HUC1 cells. (A, C, E) RT‑qPCR analysis of AQP7, AQP9, and AQP11. The results are shown as the mean ± SEM for data from four different experiments, and the expression levels were normalized against that of the housekeeping gene, GAPDH. (B, D, F) Western blot analysis was used to detect the protein expression levels of AQP7, AQP9, and AQP11. The expression levels were normalized against that of β‑actin. *P<0.05 vs. control (0 µM PPD); #P<0.05 vs. 50 µM PPD. AQP: aquaporin, SEM: standard error of the mean, RT-qPCR: reverse transcription-quantitative polymerase chain reaction, PPD: p-phenylenediamine

PPD treatment induced marked and concentration‑dependent decreases in the levels of the AQP7 transcript (Figure 4A). These findings were corroborated at the protein expression level by the results of the Western blot analysis (Figure 4B). By contrast, although the PPD‑induced changes in the expression profile of AQP9 were similar to those of AQP7, the magnitude of the decreases was found to be much lower (Figures 4C, 4D).

The expression of AQP11 is unchanged by PPD

PPD at the various concentrations investigated failed to induce any significant changes in the transcript or the protein expression level of AQP11, as shown in Figures 4E, 4F.

## Discussion

To the best of the authors’ knowledge, this is the first study to have examined the effect of arsenate and PPD on the expression of AQPs in urothelial cells. AQP3, 4, 7, 9, and 11 were all confirmed to be expressed at both the mRNA and protein levels; also, arsenate and PPD exerted differential effects on the expression of these AQPs.

The mechanisms underlying the arsenate- or PPD‑induced changes in AQP expression, and the functional significance of the alterations in AQP expression, were not explored at a deeper level in the present study. However, substantial evidence has suggested the regulatory role of AQPs in urothelial permeability [23,24]. Therefore, a reduced expression of these AQPs would be anticipated to result in an altered extracellular microenvironment, which may be perceived as a form of stress to the urothelial cell, eventually leading to functional changes in the bladder [25]. On the other hand, one of the widely accepted pathogenic mechanisms responsible for As- and PPD‑induced organ dysfunction is the excessive production of ROS. Among the known ROS, H_2_O_2_ is a crucial intracellular and intercellular signaling molecule that exerts an influence on various cellular processes, including cell differentiation, proliferation, metabolism, mobility, and so on [26]. Interestingly and importantly, peroxiporins (AQP1, 3, 5, 8, 9, and 11), the main H_2_O_2_ transporters across the cell membrane, have recently been suggested to fulfill key roles in the signal transduction pathways underlying diverse cellular functions as described above. Dysregulation of peroxiporin function can lead to oxidative stress, and eventually to cell death [27-29]. Accordingly, the reduced expression of the various AQPs observed in the present study could theoretically enhance the detrimental effects of treatment with arsenate and PPD by delaying the efflux of hydrogen peroxide. A recent study demonstrated that arsenic could induce the autophagy of keratinocytes via enhancing the expression of AQP3, suggesting a novel role of AQP3 in mediating the uptake of As, which in turn leads to cancerous skin lesions [30]. Unlike these findings in keratinocytes, however, decreased AQP3 expression was observed in arsenate‑treated urothelial cells in our study. A combination of using different cell lines, As species and concentrations, and the duration of exposure to As may, in part, explain this discrepancy.

The identification of the reduced expression levels of AQP7 and AQP9, but not of AQPs 3, 4, and 11, in PPD‑treated cells, is an interesting phenomenon that warrants further study. With regard to AQP11, neither the protein nor the mRNA expression level was significantly changed by either arsenate or PPD exposure. This is perhaps not surprising, given that it has an intracellular localization in the endoplasmic reticulum.

One of the limitations of this study is an in vitro urothelial cell culture system which excludes other components of the urothelium. It is clear that the direct effect of As or PPD on the urothelial cells is not the only mechanism involved in As- or PPD-induced urothelial damage. Another deficiency is that only a shorter period of As and PPD treatment. In practice, adverse effects of As and PPD usually manifest after chronic exposure to them.

## Conclusions

The present study was performed as a first step towards exploring the possible involvement of AQPs in As‑ and PPD‑induced bladder diseases and we demonstrated that the expressions of AQP3, 4, 7, 9, and 11 were differentially regulated by arsenate and PPD. Although the functional significance of the As- and PPD-altered expression levels of AQPs remains to be elucidated, the observations obtained in the present study suggest that AQPs might have a role in the pathogenesis of As‑ and PPD‑induced bladder diseases. In the future, in-depth in vitro and in vivo studies are both required to validate the preliminary results acquired in this study.
